# The Lingering Impact of the COVID-19 Pandemic on Colorectal Cancer Screening Modalities and Staging by Neighborhood-Level Deprivation

**DOI:** 10.14309/ctg.0000000000000991

**Published:** 2026-02-09

**Authors:** Jamie L. Romeiser, Kristin Paterson, Nicholas Allis, Joseph Valentino, Telisa Stewart

**Affiliations:** 1 Department of Public Health and Preventive Medicine, SUNY Upstate Medical University, Syracuse, New York, USA;; 2 Upstate Cancer Center, SUNY Upstate Medical University, Syracuse, New York, USA;; 3 Norton College of Medicine, SUNY Upstate Medical University, Syracuse, New York, USA;; 4 Department of Surgery, SUNY Upstate Medical University, Syracuse, New York, USA.

**Keywords:** colorectal cancer, COVID-19, early detection of cancer, FIT, social determinants of health, cancer prevention, colonoscopy

## Abstract

**INTRODUCTION:**

Disruptions to colorectal cancer (CRC) screenings occurred during the COVID-19 pandemic, but lingering effects and equity of this impact remain unclear. We evaluated local patterns and trends in CRC screening and cancer staging across 4 time periods and examined disparities by neighborhood social deprivation index (SDI).

**METHODS:**

Colonoscopies, fecal immunochemical test (FIT), fecal occult blood test, and sigmoidoscopies were identified at our institution through electronic medical records from March 2018 to February 2023 (n = 27,946). Screenings were linked to zip code-level SDI and aggregated by month across 4 periods: prepandemic (March 2018-February 2020), pandemic (March 2020-February 2021), postpandemic year 1 (March 2021–February 2022), and postpandemic year 2 (March 2022–February 2023). Analysis of variances compared monthly screening volumes and proportions from high-SDI areas across periods; χ^2^ tests assessed differences in cancer staging.

**RESULTS:**

CRC screenings dropped during March–May 2020 but rebounded, resulting in no significant difference in monthly averages across periods (*P* = 0.52). However, colonoscopies declined, whereas FIT use increased over time. The proportion of patients from high-SDI neighborhoods declined from 32.2% prepandemic to 28.9% by postpandemic year 2 (*P* = 0.002), largely driven by reduced FIT screenings among individuals from these areas (30.9%–23.7%, *P* < 0.0001). Later-stage diagnoses increased during the pandemic and postpandemic year 1 but returned to baseline by year 2.

**DISCUSSION:**

Despite volume recovery, shifts in modality use and declining representation from high-SDI neighborhoods suggest growing disparities in CRC screening. Targeted outreach is needed to understand and address unmet screening needs, to support equitable prevention efforts moving forward.

## INTRODUCTION

Colorectal cancer (CRC) remains a leading public health concern in the United States. It is the third most diagnosed cancer and the second leading cause of cancer-related deaths among both men and women nationally, including in New York State (1). Like many cancers, survival rates for CRC are highly dependent on early detection. Multiple screening modalities, including colonoscopies and stool-based tests, have been instrumental in the early detection of precancerous polyps and early signs of CRC. Because of screening advancements, incidence and mortality rates for CRC have significantly improved (2). In fact, it is estimated that well over 60% of CRC deaths may be prevented with regular and up-to-date screening (3).

However, the COVID-19 pandemic temporarily disrupted healthcare services including screenings for CRC (4). In the early months of the pandemic, a national US study reported that CRC screening volume decreased by approximately 85% compared with prepandemic levels (5). Other studies documented similar but less severe initial reductions (6), with varying recovery times observed by late 2020 into 2021 (7–10). These disruptions raised concerns about delayed diagnoses and the potential for increased late-stage cancer presentations (11).

In response to rising early onset colorectal cancers and emerging screening gaps, the US Preventive Services Task Force updated screening guidelines in 2021. New guidelines now recommend that all adults with average risk levels for CRC should begin routine screening at age 45 (12). Despite these guidelines, disparities in screenings still persist. Theories such as the Anderson model for healthcare use have been used to explain these disparities, and categorize key determinants of screening behaviors into: predisposing factors such as sociodemographic characteristics; perceived or actual need factors such as health history, beliefs, or symptoms; and enabling factors such as insurance or neighborhood characteristics (13).

Neighborhoods can either support or impede access to health care, and play a complex role in the likelihood of cancer screening (14). Several studies have shown that individuals living in areas with higher levels of social deprivation or lower socioeconomic status are less likely to adhere to CRC screening guidelines (15–18). These patterns often reflect the broader influence structural inequalities can have on cancer prevention services. Healthcare access, transportation barriers, income or housing instability, and health literacy are often reflected in measures of neighborhood deprivation, and all can play a role in cancer screenings and higher mortality rates (19,20).

The longitudinal extent to which the pandemic may have further exacerbated neighborhood disparities in screenings is not fully understood. Neighborhoods with a higher level of deprivation experienced a greater burden of hardships during the COVID-19 pandemic (21,22). Although previous studies have highlighted the short-term declines in CRC screening by neighborhood (8,23), few studies have examined longitudinal trends across different screening modalities and neighborhood characteristics, extending beyond the first year after the pandemic. Therefore, our study examines: (i) the extent to which the pandemic influenced the shift from invasive procedures such as colonoscopies to noninvasive tests such as fecal immunochemical test (FIT), and whether these shifts have persisted well into the postpandemic period, (ii) whether changes in screening practices have differentially affected various demographic groups, particularly by neighborhood deprivation, and (iii) if there are differences in cancer staging patterns across time periods and by neighborhood characteristics.

## MATERIALS AND METHODS

### Study population and data

On receiving Institutional Review Board exemption status (project number 2046821-1), deidentified data were extracted from the institutional electronic medical record. International Classification of Diseases, tenth revision diagnostic and Current Procedural Terminology codes were used to identify all patients who underwent a CRC screening procedure, including colonoscopies, FIT, fecal occult blood test (FOBT), or Sigmoidoscopy, between March 2018 and February 2023. Corresponding information on age, sex, race/ethnicity, geographic area, and the Charlson Comorbidity Index (CCI) were also collected. Race was categorized as White, African American/Black, and other; ethnicity was captured as Hispanic/non-Hispanic. Duplicate records were removed before analysis. Separately, CRC cases from our institution were identified through the New York State Cancer Registry. Information for stage was extracted for all cases within the time period of interest.

#### Social deprivation index.

The Social Deprivation Index (SDI) was initially developed in 2012 (24), and updated several times with the most recent American Community Survey data (25). The SDI is a composite measure of area level deprivation across zip-codes. The index is composed of 7 zip-code level metrics, including: % living in poverty, % <12 years of education, % single-parent households, % living in rented housing units, % living in the overcrowded housing unit, % of households without a car, and % nonemployed adults younger than 65 years of age. We chose this index because it is calculated at the zip code-level. Zip code-level data for the 2017–2022 SDI were acquired through the Robert Graham Center by request on September 11, 2024.

SDI data were linked to the electronic medical record data by zip-code level Federal Information Processing System codes. For our analysis, we categorized areas with a high SDI if the index was >75, e.g., the 75th percentile, which indicates that a zip code is at the top quartile of deprivation within in the U.S.

#### Area Deprivation Index.

As a supplemental index, we also examined screening volumes by the 2022 Area Deprivation Index (26). The ADI is a measure created to rank neighborhoods by socioeconomic disadvantage at the state or national level, and composed of 17 elements related to education, income/employment, housing, and household characteristics. Because the authors do not guarantee validity of this index at the zip code-level, we used this index strictly as a supplemental index to examine robustness of our findings.

#### Rural urban commuting area codes.

2010 Rural Urban Commuting Area Codes (RUCA) data from the USDA (27) were linked at the zip code-level, as the 2020 RUCA data were not available at the time of this analysis. Metropolitan classifications were derived using primary RUCA code guidelines, and operationalized as rural/small town, micropolitan, and urban (metropolitan).

### Outcomes

Case volumes were divided into 4 periods: prepandemic (March 2018–February 2020), pandemic (March 2020–February 2021), postpandemic year 1 (March 2021–February 2022), and postpandemic year 2 (March 2022–February 2023). The pandemic time period of March 2020 through February 2021 follows the CDC pandemic timeline of time from the start of the pandemic to roughly when the broader shutdown and gathering restrictions had eased (28). There were no differences in volumes or case make-up between 2018 and 2019. Therefore, to create the most stable estimate for the prepandemic baseline, monthly case volumes for 2 years were averaged (e.g., March 2018 and March 2019 case volume were averaged for a prepandemic March volume). The primary outcome was total average monthly CRC screening volume within the 4 periods of interest. Secondary outcomes included average monthly screening procedure volumes for colonoscopies, FIT, FOBT, and sigmoidoscopies.

For those with cancer staging data available, we examined stage categorized as 0–1, 2, 3, or 4, and early (0, 1, 2) or late (3, 4). Of note, not all cases extracted from our institutional cancer registry received their initial diagnosis at our institution.

### Statistical analysis

Missing data were minimal (total 769/27,946 = 2.75% missing data for any of the variables examined), and was therefore ignored. Screening data were aggregated by month. Because screening data were primarily analyzed on the aggregate level, most data (e.g., demographic data, types of screening tests) are described and analyzed as proportions of the total volume with 95% confidence intervals (CI). Continuous data (e.g., age, CCI) are described as means (95% CI). Volume data for the screening tests are described as mean monthly counts (95% CI). Data were compared across the 4 pandemic eras by analysis of variance tests and generalized linear models were used to compare each time period with baseline. Deaggregated data were analyzed at the individual level in a multinomial logistic regression with repeated measures to assess the odds of screening during the pandemic, postpandemic year 1, and postpandemic year 2 compared with baseline, for neighborhood SDI after adjusting for covariates.

Cancer stage, pandemic time period, and neighborhood SDI were examined using χ^2^ tests. All analyses were performed in SAS 9.4 © (Cary, NC) at the 0.05 level of significance.

## RESULTS

**Figure 1.**
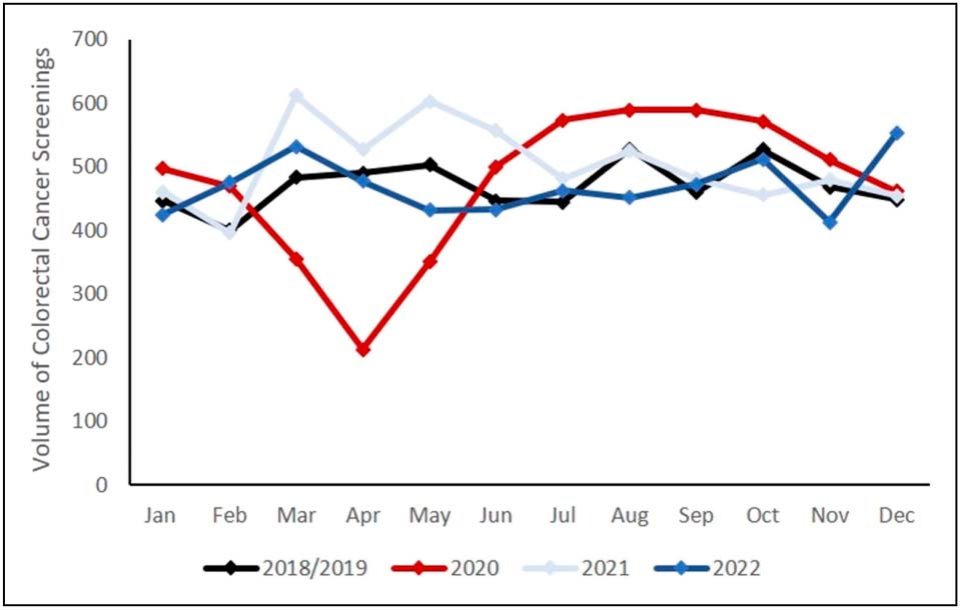
Monthly volume of all colorectal cancer screenings: During the pandemic year (2020), screening volume dipped from March-May but rebounded by July. Screening volumes normalized again in the 2 years after the pandemic.

**Figure 2.**
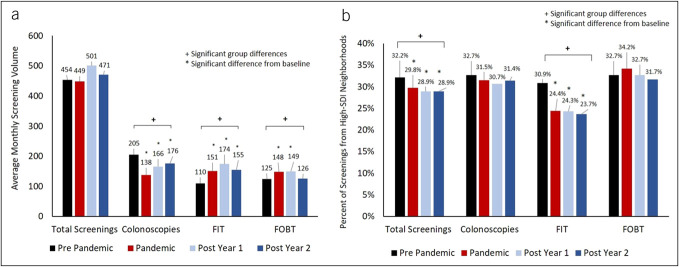
Average monthly volume and percent of screenings from high-SDI neighborhoods over time: (**a**) Total monthly volume did not differ over the 4 time periods, but colonoscopies significantly declined, whereas noninvasive screening tests increased. (**b**) Proportion of cases from high-SDI neighborhoods significantly declined over time, which was driven by changes in case composition for FIT screenings. FIT, fecal immunochemical test; FOBT, fecal occult blood test; SDI, social deprivation index.

A total of 27,946 CRC screenings were extracted and aggregated into monthly volume estimates. In the prepandemic period, there were 5,445 total CRC screenings (averaged volume of 2018 and 2019), 5,389 during the pandemic, 6,015 in postpandemic-year 1, and 5,652 in postpandemic year 2. Monthly volume of all CRC screenings dipped in March through May of 2020 compared with other years, but rebounded above and beyond baseline from July through October 2020 (Figure 1). Volumes normalized again in the 2 years after the pandemic. As a result, although the average monthly volume declined slightly in the pandemic period, all 3 time periods were not significantly different from prepandemic levels (overall *P* value = 0.24) (Figure 2a).

However, the volume and composition of the specific types of CRC screening modalities shifted over time (Figure 2a). Compared with prepandemic levels, average monthly volume of colonoscopies declined from 205/mo at baseline to 138/mo during the pandemic, which was 32.7% decline (*P* < 0.0001). Average monthly volume remained significantly lower in both of the postpandemic years (down 19% in postpandemic year 1 [*P* < 0.01], and 14.2% in postpandemic year 2 [ *P* = 0.04]). By contrast, average monthly volume of noninvasive screening tests rose. Compared with prepandemic baselines, use of FIT test use grew by 37.3% during the pandemic era (110–151, *P* < 0.0001), and continued to grow over the next 2 years compared with baseline levels (up 58.2% in post pandemic year 1, and 40.9% in post pandemic year 2, both *P* < 0.0001). FOBT screenings significantly rose but eventually returned to baseline levels.

Although total volume of screenings did not change over time, case composition did. The proportion of monthly CRC screenings in individuals from highly disadvantaged neighborhoods significantly and steadily declined over time, from 32.2% prepandemic down to 28.9% postpandemic year 2 (overall *P* = 0.002). Much of this change was not driven from differences in case composition of colonoscopies (*P* > 0.05), but from case composition differences in FIT tests. Prepandemic, 30.9% of FIT screenings were from high SDI areas. This steadily and significantly declined down to 23.7% by postpandemic year 2 (overall *P* < 0.001) (Figure 2b).

Additional aggregated information on case distributions and proportion of cases by demographic and clinical characteristics are presented by time period in Table 1. Over time, the average age of cases increased from 56.3 years in the prepandemic period to 59.1 years in the postyear 2 period (*P* < 0.001), as did the proportion of cases that were women (50.4%–55.0%, *P* = 0.001). Average CCI score stayed roughly the same over time, as did the proportion of Hispanic patients screened, but the proportion of Black patients screened declined over time (16.7%–13.2%, overall *P* value = <0.001). The proportion of screenings from rural and micropolitan areas slightly rose during the pandemic but fell again at postpandemic year 1, whereas the proportion of screenings from urban declined during the pandemic, but rose again by postpandemic year 1. Finally, robustness checks using the ADI found similar results to SDI, with lower average scores in the postpandemic periods.

**Table 1.** Aggregate monthly screening volume and proportion of cases by time period: Data were examined at the aggregate level using ANOVA tests for group comparisons and generalized linear models to compare against baseline levels

	Baseline March 2018/2019 (averaged) –Feb 2019/2020 (averaged)	Pandemic Mar 2020–Feb 2021	Postpandemic Y1 March 2021–Feb 2022	Postpandemic Y2 March 2022–Feb 2023	Group *P* value	Pandemic vs baseline *P* value	PPY1 vs baseline *P* Value	PPY 2 vs baseline *P* value
Total volume of cases	5,721	5,568	6,073	5,713				
Demographics								
Age (mean, 95% CI)	56.3 (55.9, 56.6)	57.7 (57.3, 58.1)	58.7 (57.7, 59.7)	59.1 (58.2, 60.0)	**<0.001**	**0.003**	**<0.001**	**<0.001**
CCI (mean, 95% CI)	2.6 (2.5, 2.6)	2.7 (2.6, 2.8)	2.6 (2.5, 2.7)	2.7 (2.6, 2.8)	0.3	0.1	0.47	0.11
Race/ethnicity								
White	72.9% (72.0%, 73.9%)	75.7% (74.0%, 77.3%)	76.7% (75.0%, 78.3%)	77.3% (75.9%, 78.8%)	**0.001**	**0.006**	**0.002**	**<0.001**
Black	16.7% (15.9%, 17.5%)	14.4% (13.3%, 15.6%)	13.6% (12.2%, 15.1%)	13.2% (11.9%, 14.6%)	**0.003**	**0.006**	**0.003**	**<0.001**
Other	10.4% (9.9%, 10.9%)	9.9% (8.8%, 11.0%)	9.7% (8.6%, 10.7%)	9.4% (8.5%, 10.3%)	0.39	0.39	0.22	0.1
Hispanic	5.7% (5.2%, 6.2%)	5.1% (4.3%, 5.9%)	5.5% (4.9%, 6.1%)	5.4% (4.9%, 5.9%)	0.54	0.15	0.51	0.46
Sex								
Female	50.4% (48.7%, 52.0%)	54.6% (52.4%, 56.7%)	55.8% (53.9%, 57.7%)	55.0% (53.1%, 56.8%)	**0.0003**	**0.001**	**<0.0001**	**0.0005**
Male	49.6% (47.9%, 51.3%)	45.4% (43.3%, 47.6%)	44.2% (42.3%, 46.1%)	45.0% (43.2%, 46.9%)				
Rurality								
Rural	6.5% (5.6%, 7.4%)	8.2% (7.2%, 9.2%)	7.1% (5.9%, 8.3%)	6.2% (5.4%, 6.9%)	**0.01**	**0.007**	0.3	0.66
Urban	78.0% (76.9%, 79.0%)	74.2% (72.1%, 76.2%)	76.9% (75.2%, 78.7%)	79.6% (77.8%, 81.3%)	**0.001**	**0.001**	0.35	0.15
Micropolitan	15.6% (14.7%, 16.5%)	17.6% (16.1%, 19.1%)	15.9% (14.9%, 17.0%)	14.2% (12.6%, 15.8%)	**0.003**	**0.02**	0.66	0.12
Deprivation indices								
Social deprivation index (mean, 95% CI)	56.1 (55.1, 57.1)	54.64 (53.4, 55.9)	53.7 (52.6, 54.7)	52.3 (51.5, 53.1)	**<0.001**	**0.03**	**0.0006**	**<0.001**
State area deprivation index (mean, 95% CI)	8.8 (8.8, 8.9)	8.8 (8.8, 8.9)	8.7 (8.7, 8.8)	8.8 (8.7, 8.8)	**0.0005**	0.59	**0.0009**	**0.0013**
National area deprivation index (mean, 95% CI)	72.76 (72.21, 73.31)	72.7 (72.0, 73.3)	71.6 (71.0, 72.3)	71.6 (71.2, 71.9)	**0.002**	0.76	**0.003**	**0.003**
Total CRC screenings								
Average monthly volume	453.8 (435.6, 471.9)	449.1 (378.5, 519.6)	501.3 (464.6, 537.9)	471.0 (443.4, 498.6)	0.24	0.87	0.09	0.54
Prop. High SDI	32.2% (30.7%, 33.6%)	29.8% (28.3%, 31.3%)	28.9% (27.7%, 30.2%)	28.9% (27.2%, 30.6%)	**0.004**	**0.02**	**0.002**	**0.002**
Screening types								
Colonoscopies								
Proportion of total cases	45.2% (42.6%, 47.7%)	29.4% (24.5%, 34.3%)	33.0% (30.5%, 35.4%)	37.5% (34.6%, 40.4%)	**<0.0001**	**<0.0001**	**<0.0001**	**<0.0001**
Average monthly volume	205.0 (189.0, 221.1)	137.8 (103.9, 171.8)	166.0 (145.2, 186.8)	175.8 (162.7, 189.0)	**0.0004**	**<0.0001**	**0.01**	**0.049**
Prop. High SDI	32.7% (30.7%, 34.6%)	31.5% (28.8%, 34.3%)	30.7% (28.5%, 32.9%)	31.4% (27.8%, 34.9%)	0.72	0.52	0.26	0.45
FIT								
Proportion of total cases	24.2% (23.0%, 25.5%)	34.0% (29.9%, 38.1%)	34.8% (32.7%, 36.9%)	32.9% (31.0%, 34.7%)	**<0.0001**	**<0.0001**	**<0.0001**	**<0.0001**
Average monthly volume	109.6 (103.6, 115.7)	151.3 (124.1, 178.4)	173.6 (162.6, 184.6)	154.9 (141.7, 168.1)	**<0.0001**	**0.0004**	**<0.0001**	**0.0002**
Prop. High SDI	30.9% (29.2%, 32.6%)	24.4% (21.1%, 27.7%)	24.3% (22.8%, 25.8%)	23.7% (21.4%, 26.1%)	**<0.0001**	**<0.0001**	**<0.0001**	**<0.0001**
FOB								
Proportion of Total Cases	27.6% (25.3%, 29.8%)	33.9% (31.0%, 36.8%)	29.8% (28.4%, 31.1%)	26.7% (25.3%, 28.1%)	**<0.0001**	**<0.0001**	0.1	0.52
Average monthly volume	125.2 (116.0, 134.4)	148.1 (132.2, 163.9)	149.3 (135.9, 162.7)	126.2 (114.4, 138.0)	**0.004**	**0.008**	**0.005**	0.9
Prop. High SDI	32.7% (30.6%, 34.8%)	34.2% (31.1%, 37.3%)	32.7% (30.5%, 34.9%)	31.7% (29.1%, 34.3%)	0.52	0.37	0.99	0.56
Sigmoidoscopy								
Proportion of total cases	3.0% (2.6%, 3.5%)	2.7% (2.1%, 3.2%)	2.4% (1.8%, 3.1%)	3.0% (2.6%, 3.4%)	0.28	0.27	0.09	0.83
Average monthly volume	13.9 (11.3, 16.5)	11.9 (9.1, 14.7)	12.3 (8.8, 15.9)	14.1 (11.7, 16.5)	0.55	0.28	0.39	0.93
Prop. High SDI	28.8% (24.7%, 32.9%)	28.8% (18.5%, 39.0%)	20.7% (12.3%, 29.1%)	30.2% (25.1%, 35.3%)	0.19	0.99	0.09	0.77

Categorical data are presented as proportions (95% CI), continuous data are presented as means (95% CI). Bolded p-values indicate significance. Average age increased over time, as did proportion of screenings that were women and White. Proportion of screenings among Black/African Americans declined over time. Proportion of rural screenings rose during the pandemic, but declined again in the postperiod back to baseline levels. Deprivation indices were mostly consistent showing a decline postpandemic, which indicated a decline in screenings from people in more deprived neighborhoods.

ANOVA, analysis of variance; CCI, Charlson Comorbidity Index; FIT, fecal immunochemical test; FOB, fecal occult blood.

**Figure 3.**
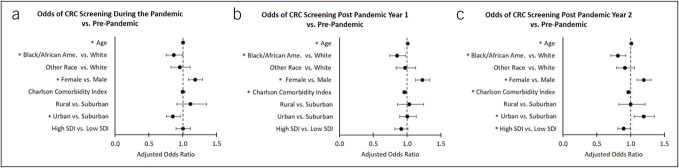
Adjusted likelihood of screening in the pandemic, postpandemic year 1, and postpandemic year 2 compared with baseline: Results from the adjusted repeated measures multinomial logistic regression are presented. Significant findings are indicated with an asterisk. After adjusting for other covariates, individuals from a high-SDI neighborhood were marginally less likely to be screened in the postpandemic year one, and significantly less likely to be screened in postpandemic year 2. CRC, colorectal cancer; SDI, social deprivation index.

Data were deaggregated and examined at the individual level in a multinomial repeated measures logistic regression. The likelihood of screening in the pandemic, postpandemic year 1, and postpandemic year 2, were compared with screening at baseline (Figure 3a–c, respectively). After controlling for various covariates, neighborhood SDI was a marginally significant negative predictor in postpandemic year 1 (odds ratio = 0.91, CI: 0.82, 1.01 [*P* = 0.09]), and a significant negative predictor of screening in postpandemic year 2 (odds ratio = 0.90, CI: 0.81, 0.99 [*P* = 0.047]) (Figure 3c). Neighborhood effects for SDI were stronger when examining FIT screenings alone, with significant adjusted effects found in the postpandemic years 1 and 2 compared with baseline (data not shown). Interestingly, race was not a significant predictor in these models.

**Figure 4.**
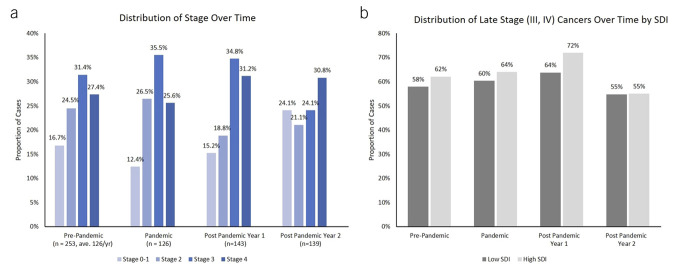
Colorectal cancer stage for all and by neighborhood SDI: (**a**) There was a slight but nonsignificant upshift in later stage cancers during the pandemic and postpandemic year 1, but this declined by postpandemic year 2. (**b**) Results were similar by neighborhood SDI. SDI, social deprivation index.

Finally, staging changes over time demonstrated some slight numeric changes, but none reached statistical significance (Figure 4a). Observationally, the volume of patients diagnosed with CRC in postpandemic year 1 increased by 13%. Staging distribution patterns shifted modestly. The proportion of stage 0–1 cases declined during the pandemic (from 16.7% to 12.4%) but rose in postpandemic year 1 (15.2%) and up to 24.1% in post pandemic year 2. The proportion of stage 2 cases remained similar in the first 2 periods, but then declined in the postpandemic years. The proportion of stage 3 diagnoses rose and then declined in postpandemic year 2, whereas stage 4 cases declined and then rose in postpandemic years 1 and 2.

When stratified by neighborhood SDI, late-stage cancers (stage III and IV) demonstrated similar patterns for both groups (Figure 4b). Numerically, the proportion of late-stage cancers was higher in the high SDI neighborhoods, but these differences were not statistically significant.

## DISCUSSION

Our findings highlight important shifts in CRC screening patterns during and after the COVID-19 pandemic. Although the total volume of CRC screenings declined sharply at the onset of the pandemic, it rebounded and remained relatively stable over the 4 time periods examined. These findings align with other local and national trends for CRC screening volumes over these time periods (8,9).

However, the composition of screening modalities changed significantly. The average monthly volume of colonoscopies declined by 32.7% during the pandemic, and did not return to baseline in subsequent years. By contrast, the use of FIT screenings increased by 37.3% during the pandemic, and remained above baseline in the subsequent years.

Despite overall recovery in total screening volumes, some known disparities were exacerbated, particularly in neighborhoods with high social deprivation. The proportion of screenings from high SDI areas declined from 32.2% prepandemic to 28.9% 2 years postpandemic. Although colonoscopy declines were parallel across SDI levels, FIT increases were not equitable, suggesting disparities in adoption of noninvasive screening.

Several factors likely contributed to the decline in colonoscopies. During the pandemic, priorities for healthcare services were shifted to mitigate the effects of COVID-19, causing nonurgent visits and elective procedures to either be canceled or delayed. This contributed to a backlog of patients waiting for CRC screening services. Fears of COVID-19 exposure and loss of insurance due to unemployment could have also played a role in the disruption of care (11), and a nationwide shortage of anesthesiologists and anesthesia providers was exacerbated by pandemic-related burnout (29). Limited availability of resources and personnel may have led healthcare systems to rely more heavily on noninvasive screening alternatives such as FIT screenings (30).

Other studies have also identified the decline in colonoscopies counterbalanced with an increase in noninvasive screening tests from prepandemic to pandemic/postpandemic time periods (31,32). The increase in FIT use may reflect the changes in screening age recommendations, increased availability, provider endorsement (33), and an increase in marketing and public messaging around noninvasive screening options such as Cologuard (34).

Yet, the uptake of FIT in high SDI areas remained disproportionately low, despite its increased use overall. This suggests ongoing inequities in screening access, health literacy, and/or reduced exposure to preventive health messaging. Continuity of care, trust in providers, and systems to support screening navigation may be less available or more costly in high-deprivation communities (35). Moreover, trust in the healthcare system was already strained in many marginalized communities before the pandemic. It is likely that trust may have further eroded during the pandemic due to misinformation, inequities in care, and vaccine hesitancy (36,37).

Although not the primary focus of our study, we also observed that the pandemic further exacerbated disparities in screenings for African Americans. This is consistent with previous work demonstrating that the pandemic disproportionately disrupted care for racial and ethnic minorities (38). We also observed an increase in the average age of individuals receiving screenings. This was unexpected given the 2021 guideline change lowering the recommended screening age to 45. National data show that screening uptake among 45-49-year-olds remained low and largely unchanged over this period, with only 21% up-to-date in 2019 compared with 20% in 2021 (39). Therefore, even though our findings show consistency with national findings, it is possible we are underserving the younger population during a time when we have a well-documented rise in young onset CRC.

Although some changes in cancer volume and stage were noted in the postpandemic years, these did not reach statistical significance. A possible explanation is the limited sample size. The decreased screening rates were also transient, and the rebound in screening may have limited the impact on cancer development and stage (40). Despite persistent disparities in screening, absolute percentage differences were small and may not translate into measurable cancer burden differences.

Our study has several strengths. First, few studies have examined changing patterns in CRC screenings beyond the first year postpandemic. We used longitudinal data from 5 years, including 2 full years after the pandemic, to examine changing trends by total screenings and by different screening modality. Second, we were able to examine both aggregated and nonaggregated data. Third, we were able perform robustness checks with other neighborhood deprivation instruments. Finally, analyses were conducted at the zip code-level, offering more granular insight than county-level studies.

Our study also has limitations. First, the analysis was conducted at a single university medical system, albeit a large and diverse institution, which may limit generalizability. Second, we were unable to calculate screening rates due to the absence of a true denominator population. Third, regional clinical practices were informally assessed and seemed stable over time. However, we cannot fully exclude the possibility of changes in our institution's catchment area, which may have influenced our findings. Finally, we were unable to fully link screening and cancer staging data because many patients were screened externally.

In conclusion, targeted research and public health interventions are urgently needed to understand underserved neighborhoods barriers and facilitators for using noninvasive screening. Outreach should focus on building collaborations and expanding outreach to high SDI neighborhoods, improve access to noninvasive tests, help patients navigate the screening process, and develop culturally tailored education campaigns to promote noninvasive screening. In addition, health systems should leverage data to monitor and address screening equity across demographic and geographic areas.

## CONFLICTS OF INTEREST

**Guarantor of the article:** Jamie L. Romeiser, PhD, MPH.

**Specific author contributions:** J.R.: Conceptualization; Investigation; Writing - original draft; Methodology; Validation; Visualization; Writing - review & editing; Software; Formal analysis; Project administration; Data curation; Supervision. K.P.: Conceptualization; Data curation; Writing—review & editing; Validation; Investigation. Nicholas Allis: Conceptualization; Data curation; Writing—review & editing; Validation; Investigation. Joseph Valentino: Conceptualization; Investigation; Methodology; Data curation; Writing—review & editing; Writing—original draft; Supervision. T.S.: Conceptualization; Investigation; Writing—original draft; Writing—review & editing; Data curation; Supervision; Methodology.

**Financial support:** None to report.

**Potential competing interests:** The authors declare no conflicts of interest.

**Study Highlights**

**WHAT IS KNOWN**

✓ Colorectal cancer screening was significantly disrupted during the early COVID-19 pandemic.
✓ Socially disadvantaged neighborhoods often have lower screening uptake and face persistent health access barriers.

**WHAT IS NEW HERE**

✓ Despite volume recovery, disparities in screening grew by neighborhood deprivation.
✓ Fecal immunochemical test screening increased overall, but not in high-deprivation neighborhoods postpandemic.

## ABBREVIATIONS:

CCI: Charlson Comorbidity Index
CRC: colorectal cancer
FIT: fecal immunochemical test
FOBT: fecal occult blood test
RUCA: Rural Urban Commuting Area Codes
SDI: social deprivation index
